# KOH Activated Carbon Coated 3D Wood Solar Evaporator with Highest Water Transport Height and Evaporation Rate for Clean Water Production

**DOI:** 10.1002/advs.202402583

**Published:** 2024-06-13

**Authors:** Mengxue Zhang, Nan Hu, Yang Guo, Wenhao Wu, Liwu Fan, Daohui Lin, Juan Wang, Kun Yang

**Affiliations:** ^1^ Department of Environmental Science Zhejiang University Hangzhou 310058 P. R. China; ^2^ Zhejiang Provincial Key Laboratory of Organic Pollution Process and Control Zhejiang University Hangzhou 310058 P. R. China; ^3^ Department of Mechanical and Aerospace Engineering Princeton University Princeton NJ 08544 USA; ^4^ Institute of Thermal Science and Power Systems School of Energy Engineering Zhejiang University Hangzhou 310027 P. R. China; ^5^ Zhejiang University‐Hangzhou Global Scientific and Technological Innovation Center Hangzhou 311200 P. R. China

**Keywords:** clean water production, KOH activated carbon, solar steam evaporator, water evaporation rate, water transport height

## Abstract

The water evaporation rate of 3D solar evaporator heavily relies on the water transport height of the evaporator. In this work, a 3D solar evaporator featuring a soil capillary‐like structure is designed by surface coating native balsa wood using potassium hydroxide activated carbon (KAC). This KAC‐coated wood evaporator can transport water up to 32 cm, surpassing that of native wood by ≈8 times. Moreover, under 1 kW m^−2^ solar radiation without wind, the KAC‐coated wood evaporator exhibits a remarkable water evaporation rate of 25.3 kg m^−2^ h^−1^, ranking among the highest compared with other reported evaporators. The exceptional water transport capabilities of the KAC‐coated wood should be attributed to the black and hydrophilic KAC film, which creates a porous network resembling a soil capillary structure to facilitate efficient water transport. In the porous network of coated KAC film, the small internal pores play a pivotal role in achieving rapid capillary condensation, while the larger interstitial channels store condensed water, further promoting water transport up more and micropore capillary condensation. Moreover, this innovative design demonstrates efficacy in retarding phenol from wastewater through absorption onto the coated KAC film, thus presenting a new avenue for high‐efficiency clean water production.

## Graphical abstract

A novel 3D solar evaporator featuring a soil capillary‐like structure is prepared by coating native balsa wood using KOH activated carbon (KAC) to address the issue of limited energy and rate efficacy for traditional 3D solar evaporator. This coating structure enables a remarkable capillary water transport height of 32 cm and a peak water evaporation rate of 25.3 kg m^−2^ h^−1^ under similar solar radiation conditions without wind.

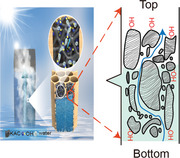

## 1 Introduction

Solar interfacial steam generation (SISG) represents a promising technology harnessing renewable solar energy to produce clean water from sources such as brine and seawater.^[^ ^1^, ^2^, ^3^, ^4^, ^5^, ^6^ ^]^ The efficiency of SISG is primarily characterized by its water evaporation rate.^[^ ^7^, ^8^ ^]^ Materials, such as wood,^[^ ^9^, ^10^, ^11^ ^]^ foam,^[^ ^12^, ^13^ ^]^ and hydrogel,^[^ ^7^, ^14^, ^15^ ^]^ are commonly employed to design 2D flat solar evaporators. However, with an ideal 100% solar thermal conversion efficiency and complete absence of heat dissipation, the theoretical maximal evaporation rate of 2D flat solar evaporators is only 1.47 kg m^−2^ h^−1^ under 1 kW m^−2^ solar radiation without wind, which fails significantly meet practical demands.^[^ ^16^, ^17^, ^18^, ^19^ ^]^ Recognizing this limitation, researchers have turned to 3D solar evaporators as a solution to enhance water evaporation rates. These 3D designs can leverage environmental heat and wind energy to augment the energy input. For example, Zhu et al.^[^ ^16^ ^]^ pioneered a 3D cylindrical solar evaporator with a projected ground area of 25.52 cm^2^. This design, utilizing a cotton core covered by a cellulose layer with a water transport height of 10 cm, achieved a water evaporation rate of 1.62 kg m^2^ h^−1^ under similar solar radiation conditions, surpassing the theoretical maximum of 2D flat solar evaporators. For 3D solar evaporators, water evaporation rate is notably influenced by the water transport height.^[^ ^16^, ^17^, ^18^, ^19^ ^]^ Increasing the water transport height from 0 to 6 cm in a reported 3D solar evaporator led to a significant enhancement in evaporation rate, from 1.28 to 2.95 kg m^2^ h^−1^ under similar solar radiation conditions.^[^ ^20^ ^]^ Up to now, the highest water transport height and evaporation rate, reported for 3D cylindrical solar evaporators under 1 kW m^−2^ solar radiation without wind, are 15 cm and 15.4 kg m^2^ h^−1^, respectively.^[^ ^21^ ^]^

**Figure 1:**
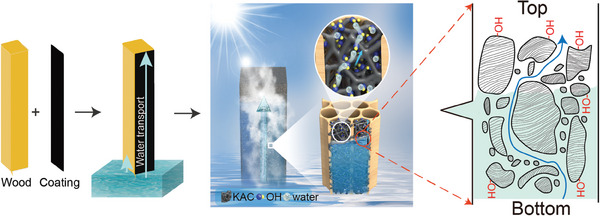
Schematic diagram of the KAC‐coated wood evaporator to facilitate the upward water transport.

The role of soil capillary force is crucial in facilitating the upward movement of groundwater to the surface soil, thereby supporting plant growth.^[^ ^22^ ^]^ Clay soil exhibits a notable capillary water transport height, often exceeding 2 meters,^[^ ^23^ ^]^ attributed to its capillary pore structure and surface hydrophilicity stemming from hydroxyl functional groups. In contrast, sandy soil demonstrates a lower capillary water transport height compared to clay, mainly due to its larger average pore radius ranging from 20 to 50 µm,^[^ ^24^ ^]^ as opposed to the narrower pore size distribution of clay ranging from 20 to 100 nm).^[^ ^25^ ^]^ Compared to capillary pore sizes of clay, the reported 3D evaporators are with relatively larger channel radii, such as ≈75,^[^ ^7^ ^]^ 2.5‐25,^[^ ^26^ ^]^ and 5–10 µm.^[^ ^27^ ^]^ According to Jurin's law,^[^ ^28^ ^]^ materials with smaller water transport channels and higher surface hydrophilicity exhibit enhanced water transport capabilities. Motivated by this understanding, we designed a 3D evaporator mimicking the capillary pore structure of clay (**Figure** **1**) by surface coating the native balsa wood using potassium hydroxide activated carbon (KAC).^[^ ^29^ ^]^ The KAC utilized in this design boasts a substantial surface area of 3315 m^2^ g^−1^, an average pore radius of 1.34 nm, and considerable hydrophilicity (with surface oxygen content reaching up to 16.98%). This KAC‐coated 3D wood solar evaporator demonstrates exceptional performance, capable of transporting water up to 32 cm and achieving an evaporate water rate up to 25.3 kg m^−2^ h^−1^ under 1 kW m^−2^ solar radiation without wind. This surpasses the previously reported highest water transport height of 15 cm and evaporation rate of 15.4 kg m^2^ h^−1^.^[^ ^21^ ^]^ Furthermore, this KAC‐coated wood evaporator exhibits the additional benefits of absorbing organic compounds, such as phenol, from wastewater, thus preventing their entry into the condensed water.

## 2 Results and Discussion

**Figure 2:**
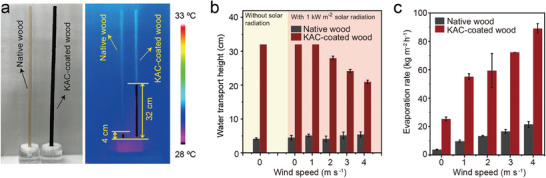
Photograph and water transport IR image without solar radiation and wind a), water transport height with or without 1 kW m^−2^ solar radiation at various wind speeds after 1 h evaporation b), and water evaporation rate with 1 kW m^−2^ solar radiation at various wind speeds c) of native wood and KAC‐coated wood evaporator.

The KAC‐coated wood evaporator demonstrates remarkable capabilities in transporting bottom water, achieving a height of up to 32 cm even without solar radiation and wind, making an eightfold increase compared to native wood evaporator (4 cm), as evidenced by lower surface temperatures observed in IR images (**Figure** **2**a). Even under 1 kW m^−2^ solar radiation and wind speeds of up to 1 m s^−1^, the KAC‐coated wood evaporator maintains its water transport height of 32 cm even after one hour of evaporation (Figure 2b; Figure S1, Supporting Information). However, with wind speed escalating to 4 m s^−1^ from 1 m s^−1^, the water transport height decreased to 21 cm (Figure 2b; Figure S1, Supporting Information). This reduction can be attributed to the higher water evaporation rate compared to the water transport rate from bottom to top within the evaporator at higher wind speeds, indicating that the water supply capacity of the KAC‐coated wood evaporator fails to maintain equilibrium with vapor escape at wind speeds exceeding 1 m s^−1^. Under 1 kW m^−2^ solar radiation and without wind, the KAC‐coated wood evaporator achieves an impressive water evaporation rate of 25.3 kg m^−2^ h^−1^ (Figure 2c), surpassing the native wood evaporator of 3.72 kg m^−2^ h^−1^ by a factor of 6.8. Notably, the observed water transport height of 32 cm and water evaporation rate of 25.3 kg m^−2^ h^−1^ for the KAC‐coated 3D wood solar evaporator in this study under 1 kW m^−2^ solar radiation and without wind, surpassing the reported highest water transport height of 15 cm and evaporation rate of 15.4 kg m^2^ h^−1^.^[^ ^21^ ^]^ In addition, with wind speed up to 4 m s^−1^, the water evaporation rate of KAC‐coated wood evaporator can up to 89.1 kg m^−2^ h^−1^ under 1 kW m^−2^ solar radiation (Figure 2c) even the water transport height decreased to 21 cm (Figure 2b). The significant increase in evaporation rate with wind speed can be attributed to the faster vapor diffusion, facilitated by the increased convective flow with higher wind speed.^[^ ^21^, ^30^ ^]^

**Figure 3:**
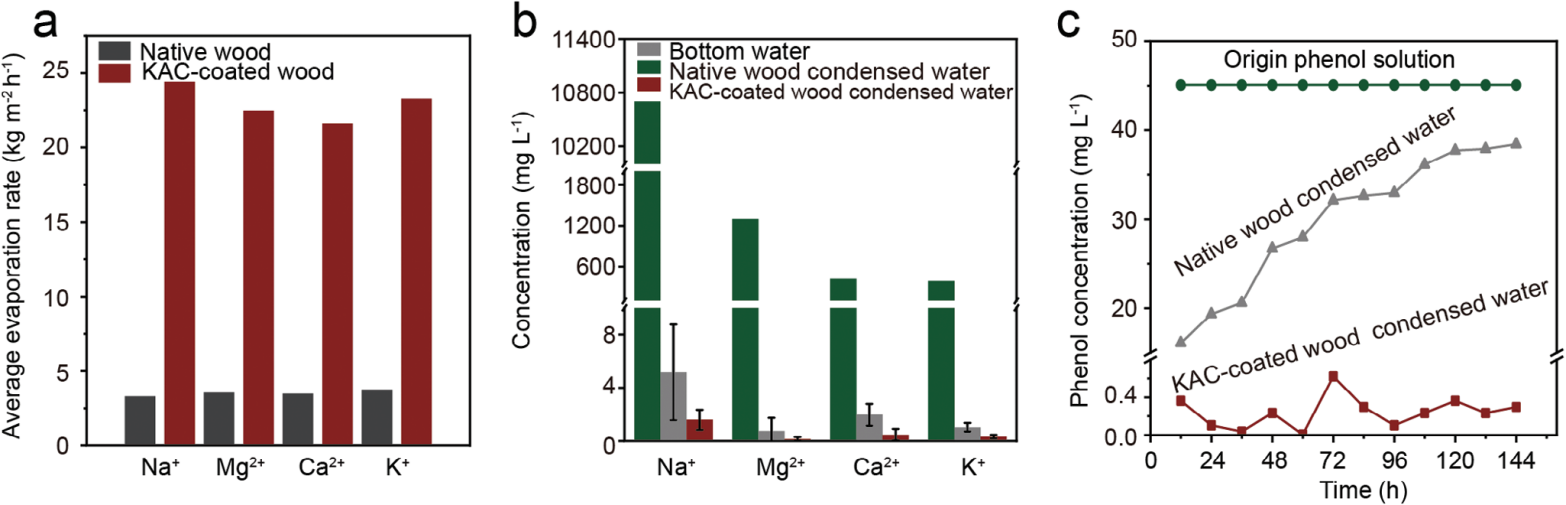
The water evaporation rate a) and the ion concentrations in condensed water b) of native wood and KAC‐coated wood evaporator in the presence of 10 700 mg L^−1^ Na^+^, 1300 mg L^−1^ Mg^2+^, 420 mg L^−1^ Ca^2+^ or 390 mg L^−1^ K^+^ in bottom water under a continuous 1 kW m^−2^ solar radiation and without wind for 24 h, and the phenol concentration in condensed water c) of native wood and KAC‐coated wood evaporator in the presence of 45 mg L^−1^ phenol in bottom water under a continuous 1 kW m^−2^ solar radiation and without wind for 144 h.

In the presence of salt ions typical of real seawater—namely, 10 700 mg L^−1^ Na^+^, 1300 mg L^−1^ Mg^2+^, 420 mg L^−1^ Ca^2+^ or 390 mg L^−1^ K^+^, the water transport height of KAC‐coated wood evaporator experiences a slight decrease under 1 kW m^−2^ solar radiation and without wind. Specifically, the transport height decreases from 32 to 29.6 cm (Na^+^), 27.0 cm (Mg^2+^), 26.6 cm (Ca^2+^), and 28.8 cm (K^+^), respectively (Figure S2, Supporting Information), which still maintaining a level ≈7 times higher than that of the native wood (4 cm in Figure 2a). Correspondingly, the water evaporation rates of the KAC‐coated wood evaporator exhibit slight decreases from 25.3 kg m^−2^ h^−1^ to 24.4 (Na^+^), 22.5 (Mg^2+^), 21.6 (Ca^2+^), and 23.3 (K^+^) kg m^2^ h^−1^, respectively (**Figure** **3**a; Figure S3, Supporting Information), still achieving rates ≈5 times higher than those of the native wood (Figure 3a; Figure S3, Supporting Information). These findings suggest excellent long‐term stability for brine treatment. Moreover, the concentrations of Na^+^, Mg^2+^, Ca^2+^, and K^+^ in the collected condensed water from KAC‐coated wood evaporator (1.61, 0.16, 0.48, and 0.34 mg L^−1^, respectively) are notably lower than those from native wood evaporator (5.16, 0.76, 1.98, and 1.05 mg L^−1^, respectively) (Figure 3b). The Na^+^ concentrations in the collected condensed water by evaporators are significantly lower than the drinking water standard established by World Health Organization (200 mg L^−1^),^[^ ^31^, ^32^ ^]^ while the concentrations of Mg^2+^ and Ca^2+^ are much lower than those typically found in Asian drinking water (i.e., <20 mg L^−1^ for Mg^2+^ and 2–80 mg L^−1^ for Ca^2+^).^[^ ^31^, ^32^ ^]^ Additionally, the KAC‐coated wood evaporator also demonstrates the potential to retard organic contaminants during the evaporation process. The phenol concentrations in collected condensed water from the bottom phenol solution (45 mg L^−1^) are reduced to below 0.6 mg L^−1^—a removal rate of 99.9%—after continuous exposure to 1 kW m^−2^ solar radiation for 144 h (Figure 3c). Conversely, the phenol concentrations in collected condensed water from the native wood evaporator increase over time and surpass those in the water from the KAC‐coated wood evaporator (Figure 3c). This effective reduction in phenol concentration by the KAC‐coated wood evaporator is attributed to phenol adsorption on the coated KAC,^[^ ^29^ ^]^ owing to the substantially larger specific surface area (3315 m^2^ g^−1^) and pore volume (2.21 m^3^ g^−1^) of KAC (Table S1; Figure S4, Supporting Information), in contrast to the relatively limited surface area (11 m^2^ g^−1^) and pore volume (0.007 m^3^ g^−1^) of native wood (Table S1; Figure S4, Supporting Information). Large surface area of KAC give the potential to adsorb more organic molecules and commonly result in a larger adsorption capacity.^[^ ^29^ ^]^ Pore filling is the underlying mechanism responsible for the adsorption of phenol by KAC.^[^ ^33^ ^]^ There is a stronger adsorption force between organic contaminant molecules and adsorbent surface sites in micropores than that in bigger pores, following the Polanyi theory and showing higher adsorption affinity.^[^ ^29^ ^]^ KAC is with 59.7% micropores (Table S1, Supporting Information), indicating the stronger adsorption force and excellent affinity for phenol. In addition, the mesopores and macropores in KAC can provide low‐resistance channels for the internal transport of phenol and improve the accessibility of micropores for phenol.

**Figure 4:**
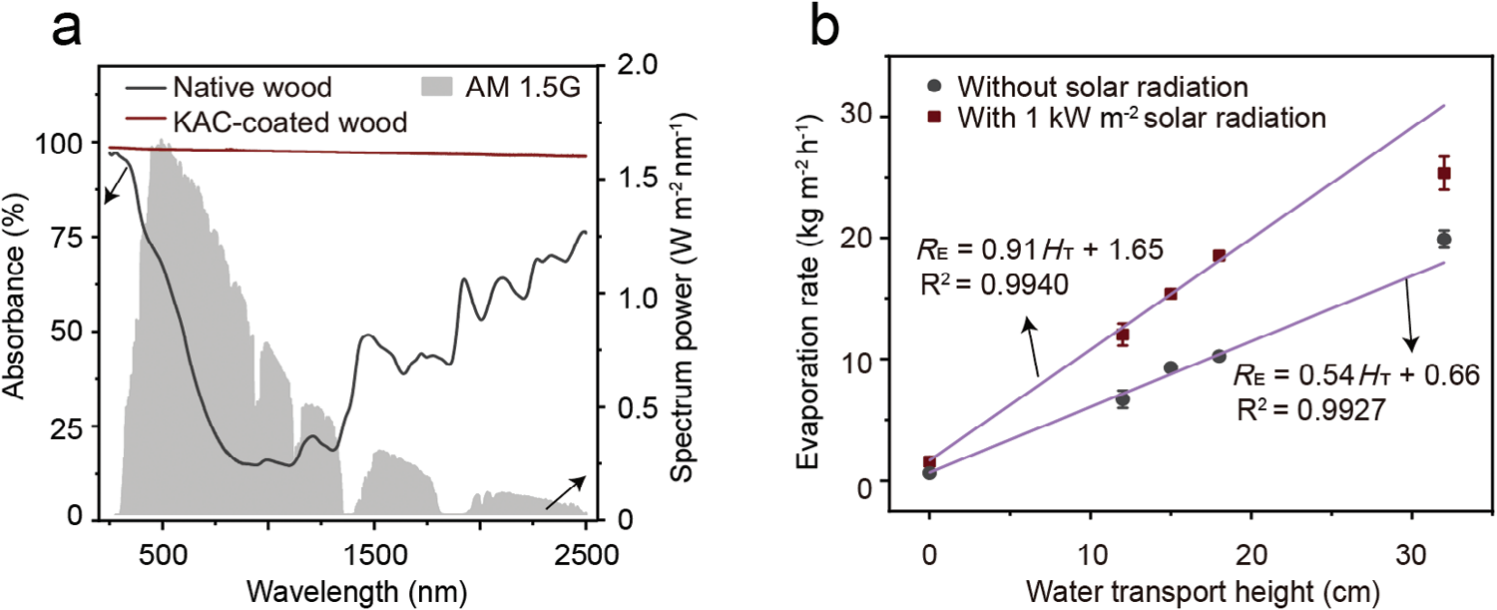
Solar absorbance spectra of native wood and KAC‐coated wood evaporator a) and the water evaporation rate versus various water transport heights of KAC‐coated wood evaporator with 1 kW m^−2^ solar radiation or without solar radiation at 0 m s^−1^ wind speed b).

The significantly higher water evaporation rate achieved by the KAC‐coated wood evaporator (25.3 kg m^−2^ h^−1^) compared to the native wood evaporator (3.72 kg m^−2^ h^−1^) can be attributed to two main factors. First, the KAC exhibits a superior solar absorption efficiency of up to 95% across the broad solar spectrum ranging from 200 to 2500 nm, as opposed to the 41.65% efficiency of native wood (**Figure** **4**a). The solar absorption efficiency of KAC is comparable with commonly used carbon‐based photothermal materials, including CNT modified filter paper of 90%,^[^ ^34^ ^]^ RGO‐SA‐CNT aerogels of 83%,^[^ ^35^ ^]^ porous rGO layer of 90%,^[^ ^36^ ^]^ graphene membrane of 93%,^[^ ^37^ ^]^ carbonized mushrooms of 79%,^[^ ^38^ ^]^ carbon cloth of 94%^[^ ^39^ ^]^ and carbon sponges of 95%.^[^ ^40^ ^]^ Illustrated by the KAC‐coated wood 2D evaporator with a flat size of 4 cm x 4 cm, a higher surface temperature of the evaporator tested by the IR images (Figure S5a, Supporting Information) can be obtained, caused by the improved solar absorption efficiency of KAC coating (Figure 4a), which leading a higher water evaporation rate (0.96 kg m^−2^ h^−1^) than that without KAC coating (0.60 kg m^−2^ h^−1^, Figure S5b, Supporting Information). Second, the KAC‐coated wood evaporator boasts a substantially greater water transport height, reaching 32 cm, in contrast to the 4 cm height observed in the native wood evaporator (Figure 2a). Furthermore, a linear relationship between the water evaporation rate (*R*_E_) and the water transport height (*H*_T_) is evident for the KAC‐coated wood evaporator at various heights (0.001, 12, 15, 18, and 32 cm) under conditions of 0 m s^−1^ wind speed with 1 kW m^−2^ solar radiation, as demonstrated by equations provided (Equation 1, Figure 4b; Figure S6, Supporting Information), consistent with findings from previous studies.^[^ ^20^ ^]^ This observation underscores the pivotal role of water transport height in determining the water evaporation performance of evaporators.

(1) $$R_{E}=\;0.91\left(\pm 0.04\right)H_{T}+1.65\left(\pm 0.53\right)$$

(2) $$R_{E}=\;0.54\left(\pm 0.03\right)H_{T}+0.66\left(\pm 0.43\right)$$

The increased water transport height of evaporators translates to a larger exposed evaporator area available for water evaporation, thereby enhancing the overall evaporation rate. In this study, the exposed evaporator area of the KAC‐coated wood evaporator varied with heights, with values from 1.0 cm^2^ (at 0.001 cm height), 17.0 cm^2^ (at 4 cm height), 49.0 cm^2^ (at 12 cm height), 61.0 cm^2^ (at 15 cm height), 73.0 cm^2^ (at 18 cm height), and 129.0 cm^2^ (at 32 cm height), respectively. Moreover, the increased water transport height of evaporators also means a greater exposed area for solar energy absorption, as illustrated in Figure 4a. This expanded surface area allows for enhanced absorption of solar energy and environmental heat energy, a phenomenon confirmed by lower temperature observed on the surface of the KAC‐coated wood evaporator compared to the surrounding temperature in IR images (Figure 2a; Figure S1, Supporting Information).^[^ ^16^, ^41^ ^]^ These factors collectively contribute to boosting the evaporation rate of the system.

(3) $$Q\;=\dot{m}\;h_{lv}\;A_{proj}=Q_{t}\;+Q_{s}$$

where *Q* is the obtained total energy required for evaporating water, $\dot{m}$ is the evaporation rate of a KAC‐coated wood evaporator at the given height under 1 kW m^−2^ solar radiation without wind (Figure 4b), *h*_lv_ is the enthalpy of phase change of water from liquid to vapor,^[^ ^2^ ^]^ *A*_proj_ is the projected area of KAC‐coated wood evaporator, *Q*_t_ is the obtained environment heat energy and *Q*_s_ is the obtained solar energy. According to the findings from the previous study,^[^ ^21^ ^]^ the obtained environment heat energy (*Q*_t_) under 1 kW m^−2^ solar radiation without wind can be analyzed by the obtained environment heat energy without solar radiation and wind (Equation 4).

(4) $$Q_{t}=\ddot{m}\;h_{lv}A_{proj}$$

where *h*_lv_ is the enthalpy of phase change of water from liquid to vapor,^[^ ^2^ ^]^ *A*_proj_ is the projected area of the KAC‐coated wood evaporator, and $\ddot{m}\;$ is the evaporation rate of a KAC‐coated wood evaporator without solar radiation and wind (Figures 4a and S6b, Supporting Information). The *Q*_t_ values of the KAC‐coated wood evaporator at various heights (0.001, 4, 12, 15, 18, and 32 cm) are 0.040, 0.177, 0.421, 0.584, 0.643, and 1.249 W (Table S2, Supporting Information), respectively, which are 0.40, 1.77, 4.21, 5.84, 6.43 and 12.49 times those of the obtained direct solar radiation energy (*q*_solar_, 0.1 W) (Table S2, Supporting Information), highlighting the significant contribution of environmental heat energy to the total energy obtained by the evaporator. This indicates that the increased exposed area of the KAC‐coated wood evaporator can significantly enhance heat extraction from the ambient environment by facilitating greater contact between water and air.^[^ ^16^, ^20^, ^42^ ^]^ The obtained solar energy (*Q*_s_, Equation 5) is determined by the direct solar radiation energy (*q*_solar_) and diffuse radiation energy (*q*_diffuse_).

(5) $$Q_{s}=q_{solar}\;+q_{diffuse}$$

Under solar radiation of 1 kW m^−2^ without wind, the diffuse radiation energy (*q*_diffuse_) obtained by the KAC‐coated wood evaporator at various heights (4, 12, 15, 18, and 32 cm) are 0.055, 0.234, 0.283, 0.420, and 0.241 W (Table S2, Supporting Information), respectively. Notably, these values are 0.50, 2.34, 2.83, 4.20, and 2.41 times those of the obtained direct solar radiation energy (*q*_solar_, 0.1 W), indicating efficient solar energy absorption by the evaporator with increased exposed evaporated area. Moreover, at a given water transport height of 32 cm, the water evaporation rate of a KAC‐coated wood evaporator with 1 kW m^−2^ solar radiation exhibits an obvious rise when the thicknesses of the KAC layer increases from 0 to 0.22 mm (Figure S7, Supporting Information), which could be attributed to the gradual establishment of the water transport channel and enhanced solar absorption by the KAC coating. At a given water transport height of 4 cm, the estimated water evaporation rate of a KAC‐coated wood evaporator with 1 kW m^−2^ solar radiation is calculated to be 5.29 kg m^−2^ h^−1^ (Equation 1). This rate is higher than the estimated water evaporation rate of 2.82 kg m^−2^ h^−1^ without solar radiation (Equation 2), and significantly surpasses that of the native wood evaporator (3.72 kg m^−2^ h^−1^). These results suggest the combined contribution of solar energy absorption and environment heat energy acquisition to the water evaporation process.

**Figure 5:**
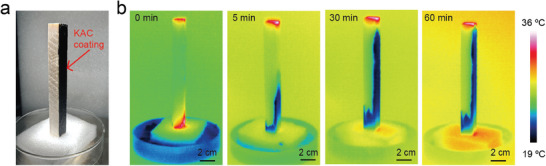
Photograph a) and water transport IR images over time b) of native wood coated with KAC on one side under 1 kW m^−2^ solar radiation and without wind.

**Figure 6:**
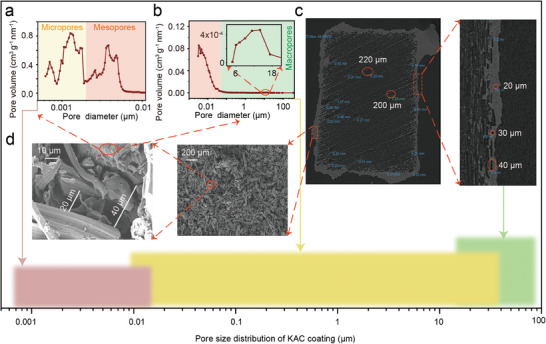
The full‐scale pore size distribution of KAC coating on native wood surface was analyzed by nitrogen adsorption method a), mercury injection methods b), X‐ray computed tomography measurement images (Slice 469/1381, c), and SEM images from a longitudinal view d).

The difference in water transport height observed between the KAC‐coated wood evaporator (32 cm) and the native wood evaporator (4 cm) depicted in Figure 2a can be attributed to the coated KAC film. This assertion is supported by the notable contrast in water transport height of the KAC‐coated side and the other sides lacking KAC coating, as illustrated in **Figure** **5**. It is hypothesized that water capillary rise governs the mechanism underlying the water transport height for 3D solar evaporators.^[^ ^17^, ^43^, ^44^, ^45^ ^]^ The capillary rise of water in 3D evaporators can be efficiently described by Jurin's law, as represented by Equation (6).^[^ ^28^ ^]^

(6) $$h_{eq}=\frac{2\sigma cos\theta }{\rho_{w}gR_{ave}}\;$$

where *h*_eq_ is the transport height of capillary water, σ is the surface tension, *θ* is the water contact angle, ρ_*w*_ is the water density, *g* is the acceleration of gravity and *R*_ave_ is the average pore radius of the evaporator (Figure S8, Supporting Information). As per Jurin's law, theoretically, evaporators characterized by a hydrophilic surface (corresponding to a larger cos*θ*) and narrow water transport channels (smaller *R*_ave_) can efficiently raise water to greater heights through capillary action (as illustrated in Figure S9, Supporting Information). Consequently, the water transport height of 3D solar evaporators is contingent upon the pore size and hydrophilicity of the evaporator.^[^ ^44^, ^46^, ^47^ ^]^ According to the provisions of the International Union of Pure and Applied Chemistry, the pores of powder materials are divided into micropores (<2 nm), mesopores (2–50 nm), and macropores (>50 nm).^[^ ^48^ ^]^ KAC particles possessed an abundance of micropores and mesopores pores, identified by the nitrogen adsorption method (**Figure** **6**a), resulting in a substantial pore volume of 2.21 m^3^ g^−1^, which surpasses that of native wood (with a pore volume of 0.007 m^3^ g^−1^, as outlined in Table S1 and Figure S4, Supporting Information). It is worth noting that, KAC exhibits a smaller pore size, characterized by an average pore radius of 1.34 nm (as detailed in Table S1 and Figure S4, Supporting Information), compared to clay (which typically ranges from 20–100 nm),^[^ ^25^ ^]^ meaning that smaller water transport channels have been successfully established. However, there is an inconsistent in water rising height with the description of Jurin's law, which could be attributed to the wide pore size distribution of KAC coating (Figure 6). The pore size distribution of KAC particles measured by the nitrogen absorption method is from <1 to ≈10 nm with an average pore radius of 1.34 nm (Figure 6a), while that measured by the mercury injection method is from <10 to ≈20 µm with an average pore radius of 36.7 nm (Figure 6b), meaning that water can exist in two forms in KAC particles, that is, film water and capillary water.^[^ ^45^ ^]^ Capillary water can flow through the pores of the evaporator,^[^ ^45^ ^]^ that is, the internal pores of KAC particles can facilitate water transport. In addition, nitrogen adsorption‐desorption isotherm and pore size distribution of KAC coating were also tested by using the native wood (1 cm × 1 cm × 0.1 cm, length x width x thickness) with KAC load mass of 1.5 mg cm^−2^. The obtained specific surface area (426 m^2^ g^−1^) and pore volume (0.30 cm^3^ g^−1^) is significantly lower than that of KAC (3315 m^2^ g^−1^ and 2.21 cm^3^ g^−1^), but much higher than that of native wood (11 m^2^ g^−1^ and 0.007 cm^3^ g^−1^, Figure S4a, Supporting Information. This indicates that KAC coating still possesses an abundance of micro‐ and meso‐ porous structure from 1 to 10 nm identified by the pore size distribution of KAC coating (Figure S4b, Supporting Information). The size of the channels between KAC particles, estimated by X‐ray computed tomography images, was ≈40 µm (Figure 6c), which was consistent with the results of the spacing between the KAC particles indicated by SEM results (Figure 6d). Therefore, the porous network of KAC coating together promotes the water transport, that is, larger interstitial channels (≈40 µm) formed by the stack of KAC particles may serve as “reservoirs”, collecting accumulated capillary water for further transport, thus enhancing the overall water transport capability (Figure S10, Supporting Information).^[^ ^49^ ^]^

**Figure 7:**
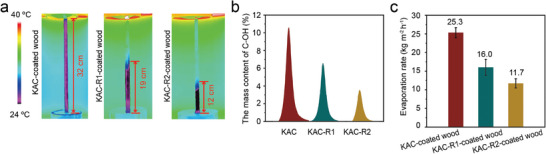
Water transport IR images a), C─OH content b), and evaporation rate c) of KAC‐coated wood, KAC‐R1‐coated wood, and KAC‐R2‐coated wood evaporator under 1 kW m^−2^ solar radiation and without wind.

KAC possesses a high degree of hydrophilicity, evidenced by its oxygen content of 16.98% and the presence of abundant oxygen‐containing functional groups such as C─OH, C═O groups, and physically absorbed water (H_2_O) on its surface (Table S3 and Figure S11, Supporting Information).^[^ ^50^ ^]^ The surface hydrophilicity of KAC is further demonstrated by the rapid disappearance of water droplet upon contact with the KAC surface, which occurs in ≈85 ms, significantly faster than on native wood surfaces (taking ≈2 s), as observed in water contact angle measurement experiments (Figure S12, Supporting Information). Through thermal annealing, the hydrophilic oxygen‐containing functional group content on KAC surface can be significantly reduced (Table S3 and Figure S11, Supporting Information), while the pore structure, morphology, and light absorption of KAC remain largely unchanged (Figures S13–S15, Supporting Information). The resulting annealed KAC, denoted as KAC‐R1 and KAC‐R2, exhibit diminished water transport heights and evaporation rates compared to the original KAC‐coated wood evaporator (**Figure** **7**a–c). This highlights the pivotal role of oxygen‐containing functional groups in determining the water transport height and evaporation rate of KAC. Therefore, the combination of abundant micro‐, meso‐ and macro‐ pores with smaller pore size and the surface hydrophilicity of KAC coating are crucial factors contributing to the rapid capillary water condensation and excellent water transport heights achieved by the KAC‐coated wood evaporator.

The robustness of the KAC‐coated wood was characterized by the particle strength tester, the maximum intensity of pressure on the transverse and longitudinal surface of the KAC‐coated wood evaporator is 2.4 and 4.1 MPa, respectively, indicating excellent mechanical strength of KAC‐coated wood evaporator (Figure S16a, Supporting Information). Although KAC‐coated wood evaporator is prepared by a simple physical coating method, the KAC particles will not fall off the wood surface even at a weed speed of 4 m s^−1^ for 30 s, which indicates that KAC particles can adhere to the wood surface steadily (Video S1, Supporting Information). The evaporation performance during a 7‐day test (under 1 kW m^−2^ solar radiation for 8 h and without solar radiation for 16 h every day) of a KAC‐coated wood evaporator was evaluated with the average daily evaporation rate remaining at 11.62 kg m^−2^ h^−1^ over 7 days, indicating the stability and recyclability of the KAC‐coated wood evaporator (Figure S16b, Supporting Information). Outdoor steam generation experiment with array of nine KAC‐coated wood evaporators was also evaluated, indicating that water evaporation rate is strongly dependent on the outdoor intensity of solar radiation and wind speed (Figure S17, Supporting Information). 457.4 g water was evaporated over 8 h, corresponding to an evaporation rate of 8.93 kg m^−2^ h^−1^, which is lower than the indoor evaporation rate (25.3 kg m^−2^ h^−1^) under the steady radiation of simulated sunlight, but still showing an excellent performance for outdoor use (Figure S17, Supporting Information). Combined with the reported results ≈3D evaporators, the reduced evaporation rate may be attributed to unsteady solar radiation and insufficient air convection upon evaporator surface caused by the array of evaporator.^[^ ^16^, ^51^ ^]^ There is no doubt that insufficient air convection is a limiting factor affecting the large‐scale application of 3D evaporators. The existing solution can be to prepare the 3D evaporator into an interconnected porous structure, and use the wind to enhance sufficient diffusion of surface vapor with a convective flow,^[^ ^30^ ^]^ which is also our follow‐up attention and research. The salt crystals formed on the surface of the evaporator are loose and porous for treating a 15wt% NaCl solution (Figure S18a, Supporting Information), which encourages the formed salt crystals to fall off the side surfaces under the action of gravity, meaning that this is a salt crystals collection process that can be continuously recycled. The corresponding evaporation rate exhibits a relative stability with an average evaporation rate of 9.6 kg m^−2^ h^−1^ (Figure S18b, Supporting Information), even with salt crystals on the surface of the evaporator, which could be attributed to the formation of loose and removable salt crystals that did not block the water transport channels. Therefore, the KAC‐coated wood evaporator is salt‐resistant.

## 3 Conclusion and Prospect

This study presents a 3D solar evaporator, consisting of KAC‐coated wood with a soil capillary‐like structure. The KAC‐coated wood evaporator achieved remarkable results, boasting the highest water evaporation rate of 25.3 kg m^−2^ h^−1^ under 1 kW m^−2^ solar radiation without wind. This outstanding performance can be attributed to two key factors: the presence of abundant oxygen‐containing functional groups and the small average pore radius of 1.34 nm in coated KAC film. These features significantly enhance the transport capability of the KAC‐coated wood, allowing for efficient utilization of ambient environment heat and solar energy, resulting in impressive evaporation rate. Importantly, the smaller average pore diameter constructed in the KAC‐coated wood serves as a barrier, preventing the entry of salt ions and organic contaminants such as phenol into the condensed water, indicating the potential of the KAC‐coated wood evaporator as a reliable and efficient solution for clean water production, surpassing existing technologies in both performance and reliability. In addition, the material of the KAC‐coated wood evaporator used in this system includes native balsa wood, polystyrene foam, and activated carbon KAC. Compared with the reported substrate materials of 3D evaporator, such as fiber,^[^ ^51^ ^]^ paper,^[^ ^18^ ^]^ foam^[^ ^52^ ^]^ and sponge,^[^ ^53^ ^]^ the substrate material of KAC‐coated wood evaporator is wood, which has strong robustness. Compared with the reported photothermal materials of 3D evaporator, such as reduced graphene oxide,^[^ ^20^ ^]^ carbon nanotubes,^[^ ^46^ ^]^ and polypyrrole,^[^ ^54^ ^]^ the photothermal material of KAC‐coated wood evaporator is activated carbon, which has the characteristics of low‐cost. Compared with the reported preparation methods of 3D evaporators, such as the freeze‐drying method,^[^ ^55^ ^]^ 3D printing method^[^ ^46^ ^]^ and hydrothermal method,^[^ ^30^ ^]^ the preparation of KAC evaporator is a physical coating method, with simple operation characteristics. The KAC‐coated wood evaporator has a disadvantage, that is, in the case of friction, the KAC will fall off the wood surface. Therefore, surface friction should be avoided during transport and usages. In the future, efforts on bonding technology should be conducted to solve this problem but not reducing the evaporation rate. Overall, the water transport strategy demonstrated by this evaporator holds promise for inspiring the development of material/structures with exceptional water transport capillary force for various other applications, and the findings of this study represent a significant advancement in the field of solar‐driven water evaporation, with potential implications for addressing global water scarcity challenges.

## 4 Experimental Section

### Materials

Native balsa wood (density: 100–120 kg cm^−3^) was bought from Zhuhai Shenmu Co., Ltd. Hydrochloric acid with purity of 36.0–38.0 wt.% and potassium hydroxide with purity of >85.0 wt.% were all purchased from Sinopharm Chemical Reagent Co., Ltd. Phenol with purity of ≥99 wt% was purchased from Hangzhou Shuanglin Chemical Co. Deionized (DI) water with a resistance of 18.2 MΩ cm was used in experiments.

### Preparation of KAC Powder

^[^ ^29^ ^]^ The purchased bamboo powder was first dried at 105 °C for 24 hours to remove the moisture content. Subsequently, the dried powder was calcinated in a furnace with a constant heating rate (5 °C min^−1^) up to 500 °C for 1 h within a nitrogen atmosphere. This calcined power was then mechanically blended with KOH powder at weight ratio of 6:1. After that, the mixture was compacted and subjected to further heating in a furnace with a constant heating rate (5 °C min^−1^) up to 900 °C for 1 h under the nitrogen atmosphere. Following this step, the calcined product was soaked in a 0.5 m HCl aqueous solution to eliminate residual KOH, followed by washing with DI water until pH of the eluent reached 6–7. The solid sample (i.e., KAC powder) was collected and dried at 105 °C for 24 h for experiments. Additionally, the KAC powder was re‐calcinated in the furnace at 500 and 1000 °C for 2 h each, within a nitrogen atmosphere, to obtain the KAC‐R1 and KAC‐R2, respectively.^[^ ^56^, ^57^, ^58^ ^]^

### Preparation of the KAC‐Coated Wood Evaporator

The purchased native balsa wood was tailored into a column structure with a size of 1.0 cm × 1.0 cm (length × width) and various heights. Subsequently, surface of these wood columns were coated using a brush with a mixture of KAC‐dispersed in ethanol. The coated wood evaporators were left to dry naturally for 24 h before being utilized in evaporation experiments. It was determined that the maximum loading amount of KAC on the wood surface was 1.5 mg cm^−2^. Estimated by X‐ray computed tomography measurement, the thickness of KAC layer with KAC load mass of 1.5 mg cm^−2^ can be identified as ≈0.22 mm (Figure S19, Supporting Information). Therefore, the thicknesses of the KAC layer was roughly estimated to be 0.04, 0.10, 0.16, and 0.22 mm based on the loaded mass of the KAC of 0.3, 0.7, 1.1, and 1.5 mg cm^−2^. KAC is coated on the surface of the block with size of 4.0 cm × 4.0 cm × 1.0 cm (length × width × height) to obtain a flat solar evaporator, and KAC is coated on the surface of the block with size of 5.0 cm × 5.0 cm × 20.0 cm (length × width × height) to test the adhesion between KAC coating and wood.

### Performance Measurement of the Evaporators

Water evaporation tests were conducted on an evaporation‐condensation device (Figure S20, Supporting Information) with a solar simulator (CEL‐AAAS50, Aulight) having an optical power meter (GEL‐FZ‐A) to calibrate the solar flux at the room temperature of 25 °C and relative humidity of ≈40%. The obtained KAC‐coated wood evaporators were placed on water surface under the support of an extruded polystyrene (EPS) for evaporation experiments. The mass loss of the water was recorded by an electronic balance (Mettler Toledo, ME802E) with an accuracy of 0.01 g. All evaporators were placed on water surface for 0.5 h to stabilize before conducting the experiment. An outdoor steam generation experiment with an array of nine KAC‐coated wood evaporators (projected area, 80 mm × 80 mm) was conducted on the roof at Zijingang Campus of Zhejiang University on 2th May 2024. Monitoring of the solar radiation, wind speed, and water mass change of KAC‐coated wood evaporators during the outdoor test from 9:00 a.m. to 5:00 p.m. Simulated wastewater (45 mg L^−1^ phenol solution) and seawater (10 700 mg L^−1^ Na^+^, 1300 mg L^−1^ Mg^2+^, 420 mg L^−1^ Ca^2+^ and 390 mg L^−1^ K^+^ solution) were also tested to evaluate performance of the evaporators for wastewater containing VOCs such as phenol and seawater. The concentration of phenol in the collected condensed water was detected by UV–vis spectrophotometer (Shimadzu, UV‐2550) at an absorption wavelength of 269 nm. The concentrations of saline ions in the collected condensed water were measured by inductively coupled plasma mass spectrometry (ICPMS, PerkinElmer NexION 300X). Wind speed was changed by placing a variable speed fan (Changhong, China) and measured by the air flow anemometer (BENETECH, China).

### Characterizations

The structure and morphologies of the evaporators were investigated by Field Emission Scanning Electron Microscopy (FESEM, Zeiss GEMINI 300, Germany) with the scanning voltage of 1 kV. The absorption spectra of evaporators were measured using a UV–vis–NIR spectrophotometer (Agilent, Cary 5000) equipped with an integrating sphere (Agilent, Internal DRA‐2500). X‐ray photoelectron spectroscopy (XPS) measurements were conducted by a VG ESCALAB MARK II using a focused monochromatic Mg Kα X‐ray radiation. Water contact angle measurement was conducted by a standard contact‐angle analyzer (OSA Optical Surface Analyzer‐OSA200‐B). The surface area and pore size distribution were calculated from N_2_ adsorption data using an AUTOSORB AS‐1 physisorption analyzer (Quantachrome) by the Brunauer‐Emmet‐Teller (BET) method and the NLDFT method, respectively. The distribution of pore size was also measured by Mercury Injection Apparatus (AutoPore IV 9510). The average pore radiuses of the water transport channels of the KAC‐coated wood evaporator were measured by X‐ray computed tomography instrument (NIKON XTH 320). The infrared radiation (IR) images were taken by a thermal imaging camera (DS‐2TD26PFT‐10/P, HIKVISION). The robustness of the KAC‐coated wood was characterized by the particle strength tester (YD‐KD3, China).

## Conflict of Interest

The authors declare no conflict of interest.

## Author Contributions

M.Z. performed conceptualization, methodology, validation, formal analysis, investigation, visualization, Wrote – original draft, review, and editing. K.Y. and J.W. performed conceptualization, methodology, validation, Wrote – review and edited, supervision, and Funding acquisition. W.W., L.F., and D.L. performed investigation.

## Supporting information

Supporting Information.

Supplemental Video 1

## Data Availability

The data that support the findings of this study are available from the corresponding author upon reasonable request.

## References

[1] L. Zhang , Z. Xu , L. Zhao , B. Bhatia , Y. Zhong , S. Gong , E. N. Wang , Passive, Environ. Earth Sci. 2021, 14, 1771.

[2] M. Gao , L. Zhu , C. K. Peh , G. W. Ho , Environ. Earth Sci. 2019, 12, 841.

[3] P. Tao , G. Ni , C. Song , W. Shang , J. Wu , J. Zhu , G. Chen , T. Deng , Nat. Energy 2018, 3, 1031.

[4] S. Cao , P. Rathi , X. Wu , D. Ghim , Y. S. Jun , S. Singamaneni , Adv. Mater. 2020, 33, 2000922. 10.1002/adma.202000922 32537817

[5] H. Ghasemi , G. Ni , A. M. Marconnet , J. Loomis , S. Yerci , N. Miljkovic , G. Chen , Nat. Commun. 2014, 5, 4449. 25043613 10.1038/ncomms5449

[6] C. Dang , Y. Cao , H. Nie , W. Lang , J. Zhang , G. Xu , M. Zhu , Nat. Water 2014, 2, 115.

[7] F. Zhao , X. Zhou , Y. Shi , X. Qian , M. Alexander , X. Zhao , S. Mendez , R. Yang , L. Qu , G. Yu , Nat. Nanotechnol. 2018, 13, 489. 29610528 10.1038/s41565-018-0097-z

[8] L. Zhang , B. Tang , J. Wu , R. Li , P. Wang , Adv. Mater. 2015, 27, 4889. 26184454 10.1002/adma.201502362

[9] C. Jia , Y. Li , Z. Yang , G. Chen , Y. Yao , F. Jiang , Y. Kuang , G. Pastel , H. Xie , B. Yang , S. Das , L. Hu , Joule 2017, 1, 588.

[10] C. Chen , Y. Li , J. Song , Z. Yang , Y. Kuang , E. Hitz , C. Jia , A. Gong , F. Jiang , J. Y. Zhu , B. Yang , J. Xie , L. Hu , Adv. Mater. 2017, 29, 1701756. 10.1002/adma.201701756 28605077

[11] Y. Kuang , C. Chen , S. He , E. M. Hitz , Y. Wang , W. Gan , R. Mi , L. Hu , Adv. Mater. 2019, 31, 1900498. 10.1002/adma.201900498 30989752

[12] Q. Jiang , L. Tian , K.‐K. Liu , S. Tadepalli , R. Raliya , P. Biswas , R. R. Naik , S. Singamaneni , Adv. Mater. 2016, 28, 9400. 27432591 10.1002/adma.201601819

[13] H. Ren , M. Tang , B. Guan , K. Wang , J. Yang , F. Wang , M. Wang , J. Shan , Z. Chen , D. Wei , H. Peng , Z. Liu , Adv. Mater. 2017, 29, 1702590. 10.1002/adma.201702590 28833544

[14] X. Zhou , Y. Guo , F. Zhao , W. Shi , G. Yu , Adv. Mater. 2020, 32, 2007012. 10.1002/adma.202007012 33184918

[15] H. Zou , X. Meng , X. Zhao , J. Qiu , Adv. Mater. 2023, 35, 2207262. 10.1002/adma.202207262 36366909

[16] X. Li , J. Li , J. Lu , N. Xu , C. Chen , X. Min , B. Zhu , H. Li , L. Zhou , S. Zhu , T. Zhang , J. Zhu , Joule 2018, 2, 1331.

[17] X. Wu , Z. Wu , Y. Wang , T. Gao , Q. Li , H. Xu , Adv. Sci. 2021, 8, 2002501. 10.1002/advs.202002501 PMC8025000 33854876

[18] Y. Wang , X. Wu , B. Shao , X. Yang , G. Owens , H. Xu , Sci. Bull. 2020, 65, 1380. 10.1016/j.scib.2020.04.036 36659217

[19] X. Xu , S. Ozden , N. Bizmark , C. B. Arnold , S. S. Datta , R. D. Priestley , Adv. Mater. 2021, 33, 2007833. 10.1002/adma.202007833 33786873

[20] Y. Wang , X. Wu , X. Yang , G. Owens , H. Xu , Nano Energy 2020, 78, 105269.

[21] C. T. K. Finnerty , A. K. Menon , K. M. Conway , D. Lee , M. Nelson , J. J. Urban , D. Sedlak , B. Mi , Environ. Sci. Technol. 2021, 55, 15435. 34739209 10.1021/acs.est.1c04010

[22] Y. Fan , H. Li , G. Miguez‐Macho , Science 2013, 339, 940. 23430651 10.1126/science.1229881

[23] W. Chesworth , M. C. Arbestain , F. Macías , O. Spaargaren , Y. Mualem , H. J. Morel‐Seytoux , Encyclopedia of Soil Science: Encyclopedia of Earth Sciences Series Ch., Springer, Dordrecht, NL 2008, *Vol*. 8.

[24] Y. Tang , J. Li , Environ. Earth Sci. 2018, 77, 657.

[25] D. Sidney , Clay. Clay. Miner. 1969, 18, 7.

[26] C. Li , D. Jiang , B. Huo , M. Ding , C. Huang , D. Jia , H. Li , C.‐Y. Liu , J. Liu , Nano Energy 2019, 60, 841.

[27] M. Gao , C. K. Peh , L. Zhu , G. Yilmaz , G. W. Ho , Adv. Energy Mater. 2020, 10, 2000925.

[28] J. Jurin , Philos. Trans. R. Soc. 1718, 30, 739.

[29] K. Yang , L. Zhu , J. Yang , D. Lin , Sci. Total Environ. 2018, 618, 1677. 29054641 10.1016/j.scitotenv.2017.10.018

[30] J. Li , X. Wang , Z. Lin , N. Xu , X. Li , J. Liang , W. Zhao , R. Lin , B. Zhu , G. Liu , L. Zhou , S. Zhu , J. Zhu , Joule 2020, 4, 928.

[31] W. H. Organization , Guidelines for Drinking‐water Quality, https://iris.who.int/bitstream/handle/10665/44584/9789241548151_eng.pdf?sequence=1&isAllowed=y, 2011 (accessed: February 2024).

[32] W. H. Organization , Calcium, and magnesium in drinking water : public health significance, https://iris.who.int/bitstream/handle/10665/43836/9789241563550_eng.pdf?sequence=1&;isAllowed=y, 2009 (accessed: February 2024).

[33] T. H. Nauyen , H. H. Cho , D. L. Poster , W. P. Ball , Environ. Sci. Technol. 2007, 41, 1212. 17593721 10.1021/es0617845

[34] P. Yang , K. Liu , Q. Chen , J. Li , J. Duan , G. Xue , Z. Xu , W. Xie , J. Zhou , Energy. Environ. Sci. 2017, 10, 1923.

[35] X. Hu , W. Xu , L. Zhou , Y. Tan , Y. Wang , S. Zhu , J. Zhu , Adv. Mater. 2016, 29, 1604031.

[36] L. Shi , Y. Wang , L. Zhang , P. Wang , J. Mater. Chem. A. 2017, 5,16212.

[37] P. Zhang , J. Li , L. Lv , Y. Zhao , L. Qu , ACS Nano 2017, 11, 5087. 28423271 10.1021/acsnano.7b01965

[38] N. Xu , X. Hu , W. Xu , X. Li , L. Zhou , S. Zhou , J. Zhu , Adv. Mater. 2017, 29, 1606762. 10.1002/adma.201606762 28520092

[39] Y. Jin , J. Chang , Y. Shi , S. Hong , P. Wang , J. Mater. Chem. A. 2018, 6, 7942.

[40] L. Zhu , M. G. , C. K. N. Peh , X. Wang , G. W. Ho , Adv. Energy. Mater. 2018, 8, 1702149.

[41] Y. Shi , R. Li , Y. Jin , S. Zhuo , L. Shi , J. Chang , S. Hong , K.‐C. Ng , P. Wang , Joule 2018, 2, 1171.

[42] J. Zhou , Y. Gu , P. Liu , P. Wang , L. Miao , J. Liu , A. Wei , X. Mu , J. Li , J. Zhu , Adv. Funct. Mater. 2019, 29, 1903255.

[43] F. Gong , H. Li , W. Wang , J. Huang , D. Xia , J. Liao , M. Wu , D. V. Papavassiliou , Nano Energy 2019, 58, 322.

[44] L. Zhu , M. Gao , C. K. N. Peh , X. Wang , G. W. Ho , Adv. Energy Mater. 2018, 8, 1702149.

[45] H. Liang , Q. Liao , N. Chen , Y. Liang , G. Lv , P. Zhang , B. Lu , L. Qu , Angew. Chem., Int. Ed. 2019, 58, 19041. 10.1002/anie.201911457 31605566

[46] Y. Li , T. Gao , Z. Yang , C. Chen , W. Luo , J. Song , E. Hitz , C. Jia , Y. Zhou , B. Liu , B. Yang , L. Hu , Adv. Mater. 2017, 29, 1700981. 10.1002/adma.201700981 28470982

[47] Y. Ito , Y. Tanabe , J. Han , T. Fujita , K. Tanigaki , M. Chen , Adv. Mater. 2015, 27, 4302. 26079440 10.1002/adma.201501832

[48] M. Thommes , K. Thommes , A. V. Neimark , J. P. Olivier , F. R. Reinoso , J. Rouquerol , K. S. W. Sing , Pure Appl. Chem. 2015, 87, 1051.

[49] J. Bachmann , R. R. van der Ploeg , J. Plant Nutr. Soil Sc. 2002, 165, 468.

[50] M. A. Ahsan , A. R. Puente Santiago , Y. Hong , N. Zhang , M. Cano , E. Rodriguez‐Castellon , L. Echegoyen , S. T. Sreenivasan , J. C. Noveron , J. Am. Chem. Soc. 2020, 142, 14688. 32786805 10.1021/jacs.0c06960

[51] C. Tu , W. Cai , X. Ouyang , H. Zhang , Z. Zhang , Small 2019, 15, 1902070. 10.1002/smll.201902070 31379088

[52] Y. Wang , X. Wu , T. Gao , Y. Lu , X. Yang , G. Y. Chen , G. Owens , H. Xu , Nano Energy 2021, 79, 105477.

[53] B. Shao , Y. Wang , X. Wu , Y. Lu , X. Yang , G. Y. Chen , G. Owens , H. Xu , J. Mater. Chem. A. 2020, 8, 11665.

[54] Y. Kong , Y. Gao , B. Gao , Y. Qi , W. Yin , S. Wang , F. Yin , Z. Dai , Q. Yue , Chem. Eng. J. 2022, 445, 136701.

[55] W. Xu , Y. Xing , J. Liu , H. Wu , Y. Cui , D. Li , D. Guo , C. Li , A. Liu , H. Bai , ACS. Nano 2019, 13, 7930. 31241310 10.1021/acsnano.9b02331

[56] X. Miao , X. Chen , W. Wu , D. Lin , K. Yang , Chem. Eng. J. 2022, 438, 135606.

[57] C. Chen , Q. Zhang , M. Yang , C. Huang , Y. Yang , M. Wang , Carbon 2012, 50, 3572.

[58] G. Hotova , V. Slovak , O. S. Soares , J. L. Figueiredo , M. F. Pereira , Carbon 2018, 134, 255.

