# Supplementary material for: KOH Activated Carbon Coated 3D Wood Solar Evaporator with Highest Water Transport Height and Evaporation Rate for Clean Water Production
Source: Adv Sci (Weinh). 2024 Jun 13;11(30):2402583. doi: 10.1002/advs.202402583 (PMC11321681; doi:10.1002/advs.202402583)
Supplement: Supplementary file 1 — Supporting Information. [file ADVS-11-2402583-s002.pdf]

ADVANCED SCIENCE
Open Access

Supporting Information

for *Adv. Sci.*, DOI 10.1002/advs.202402583

KOH Activated Carbon Coated 3D Wood Solar Evaporator with Highest Water Transport Height and Evaporation Rate for Clean Water Production

Mengxue Zhang, Nan Hu, Yang Guo, Wenhao Wu, Liwu Fan, Daohui Lin, Juan Wang and Kun Yang**

Supporting Information

KOH Activated Carbon Coated 3D Wood Solar Evaporator with Highest Water Transport Height and Evaporation Rate for Clean Water Production

Mengxue Zhang, Nan Hu, Yang Guo, Wenhao Wu, Liwu Fan, Daohui Lin, Juan Wang, and Kun Yang**

M. Zhang, Y. Guo, Prof. W. Wu, Prof. D. Lin, Prof. J. Wang, Prof. K. Yang
Department of Environmental Science
Zhejiang University
Hangzhou 310058, P. R. China
E-mail: kyang@zju.edu.cn; wjuan@zju.edu.cn

M. Zhang, Prof. W. Wu, Prof. D. Lin, Prof. K. Yang
Zhejiang Provincial Key Laboratory of Organic Pollution Process and Control
Zhejiang University
Hangzhou 310058, P. R. China

Dr. N. Hu
Department of Mechanical and Aerospace Engineering
Princeton University
Princeton, NJ 08544, USA

Prof. L. Fan
Institute of Thermal Science and Power Systems
School of Energy Engineering
Zhejiang University
Hangzhou 310027, P. R. China

Prof. K. Yang
Zhejiang University-Hangzhou Global Scientific and Technological Innovation Center
Hangzhou 311200, P. R. China

Supporting Figures, Tables

Table S1. BET surface area and pore properties of native wood, KAC, KAC-R1 and KAC-R2 particle.

Sample	SA [m^2 g^{-1}] a)	V_{total} [cm^3 g^{-1}] b)	V_{micro} [cm^3 g^{-1}] c)	R_{ave} [nm] d)
Native wood	11	0.007	0.005	1.28
KAC	3315	2.21	1.32	1.34
KAC-R1	3265	2.21	1.27	1.36
KAC-R2	3153	1.98	1.23	1.26

a)SA: BET surface area (m^2 g^{-1}) calculated from the nitrogen absorption-desorption isotherm, b)V_{total}: total pore volume (cm^3 g^{-1}) calculated by DFT method, c)V_{micro}: micropore volume (cm^3 g^{-1}) calculated by DFT method, d)R_{ave}: average pore radius (nm).

Table S2. Energy data obtained by KAC-coated wood evaporator with heights of 0.001, 4, 12, 15, 18 and 32 cm under 1 kW m^{-2} solar radiation or without solar radiation and without wind.

Height H_T (cm)	Evaporation rate R_E (kg m^{-2} h^{-1})	Phase change enthalpy h_{lv} (kJ g^{-1})	Projected area A_{proj} (cm^2)	Total energy Q (W)	Environment heat Q_t (W)	Solar radiation q_{solar} (W)	Diffuse radiation $q_{diffuse}$ (W)
0.001	1.55	2.26	1	0.097	0.040	0.100	0
4	5.29	2.26	1	0.332	0.177	0.100	0.055
12	12.03	2.26	1	0.755	0.421	0.100	0.234
15	15.40	2.26	1	0.967	0.584	0.100	0.283
18	18.53	2.26	1	1.163	0.643	0.100	0.420
32	25.33	2.26	1	1.590	1.249	0.100	0.241

Table S3. Surface chemical properties of KAC, KAC-R1 and KAC-R2 particle conducted by XPS measurement.

Sample	XPS Surface element (%)		XPS O1s structure (%)		
	C	O	C=O	C-OH	H_2O
KAC	83.02	16.98	1.97	10.65	4.36
KAC-R1	87.90	12.10	2.28	6.71	3.11
KAC-R2	90.55	9.45	3.38	3.50	2.57

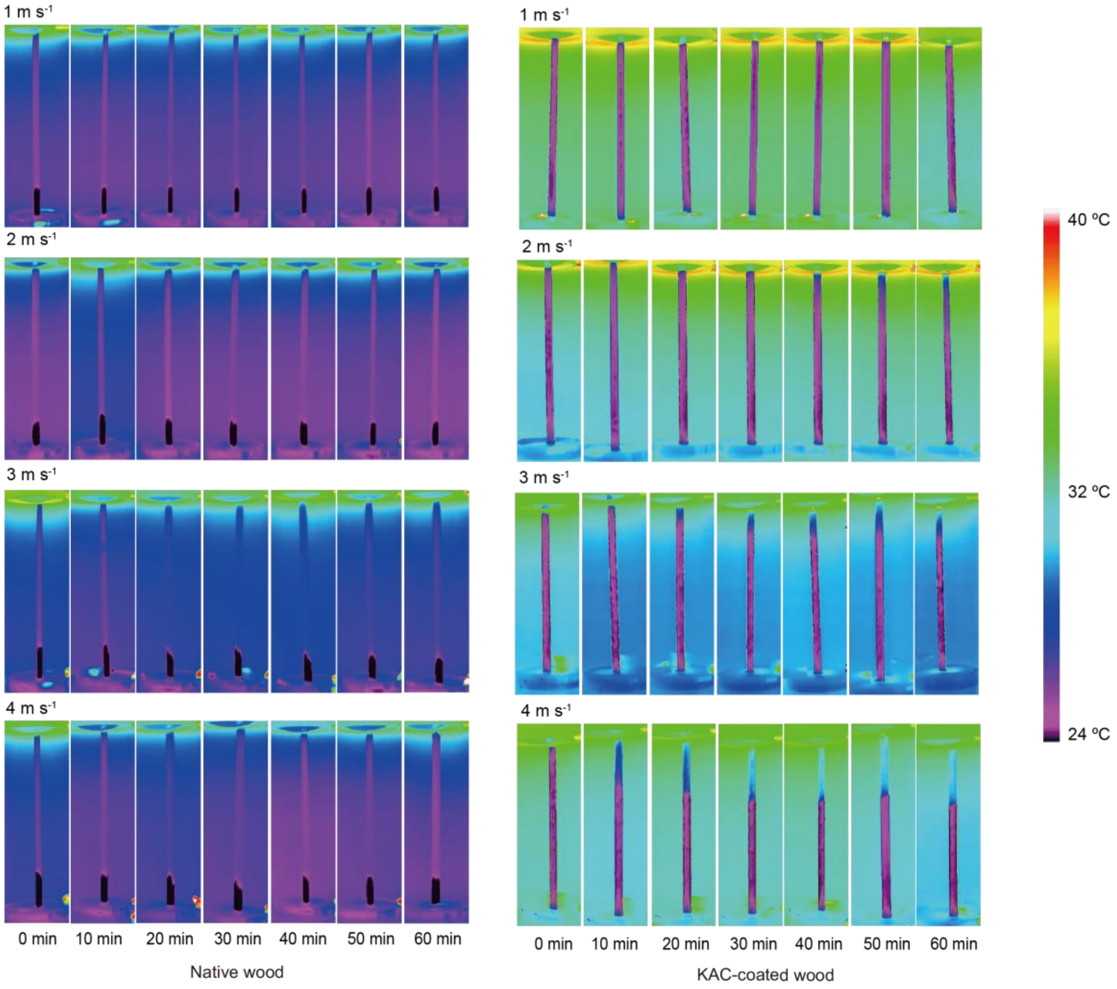

Figure S1. IR images of native wood and KAC-coated wood evaporator under 1 kW m^{-2} solar radiation and wind speeds from 1.0 to 4.0 m s^{-1}.

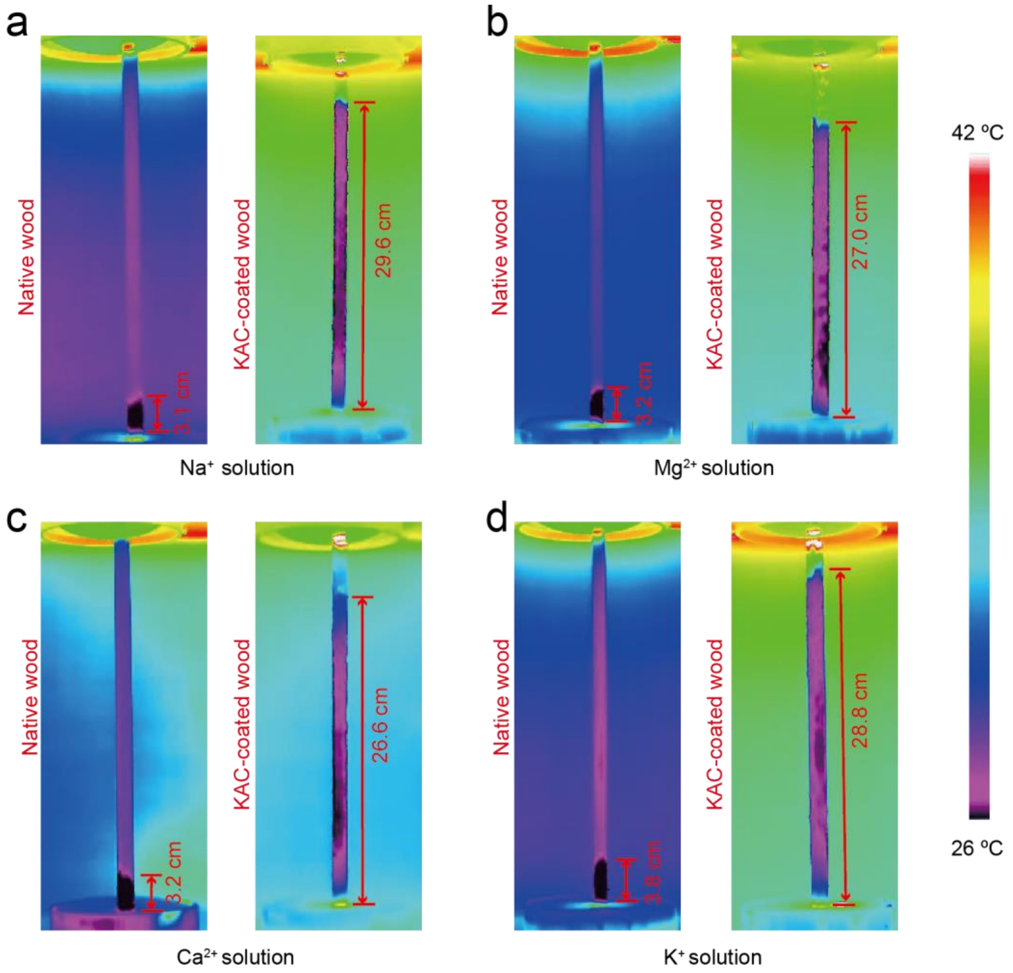

Figure S2. IR images of native wood and KAC-coated wood evaporator under 1 kW m^{-2} solar radiation and without wind in the presence of 10700 mg L^{-1} Na^+ (a), 1300 mg L^{-1} Mg^{2+} (b), 420 mg L^{-1} Ca^{2+} (c) or 390 mg L^{-1} K^+ (d) in bottom water.

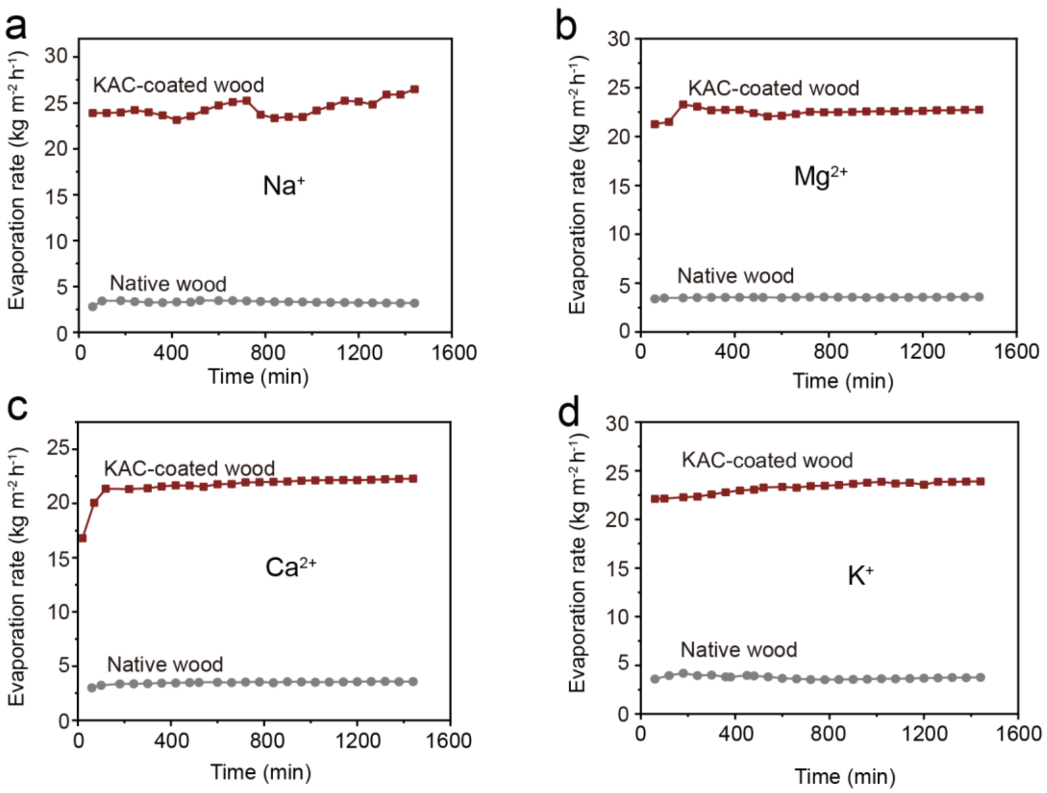

Figure S3. Water evaporation rates of native wood and KAC-coated wood evaporator under 1 kW m^{-2} solar radiation and without wind in the presence of 10700 mg L^{-1} Na^{+} (a), 1300 mg L^{-1} Mg^{2+} (b), 420 mg L^{-1} Ca^{2+} (c) or 390 mg L^{-1} K^{+} (d) in bottom water.

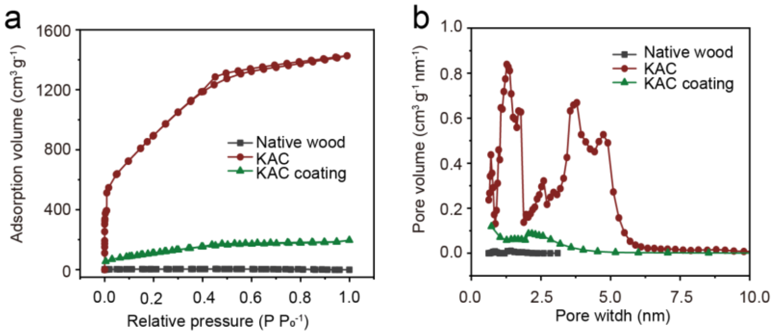

Figure S4. N_2 adsorption-desorption isotherms at 77 K (a) and pore size distributions calculated from isotherms by NLDFT method (b) of native wood, KAC and KAC coating.

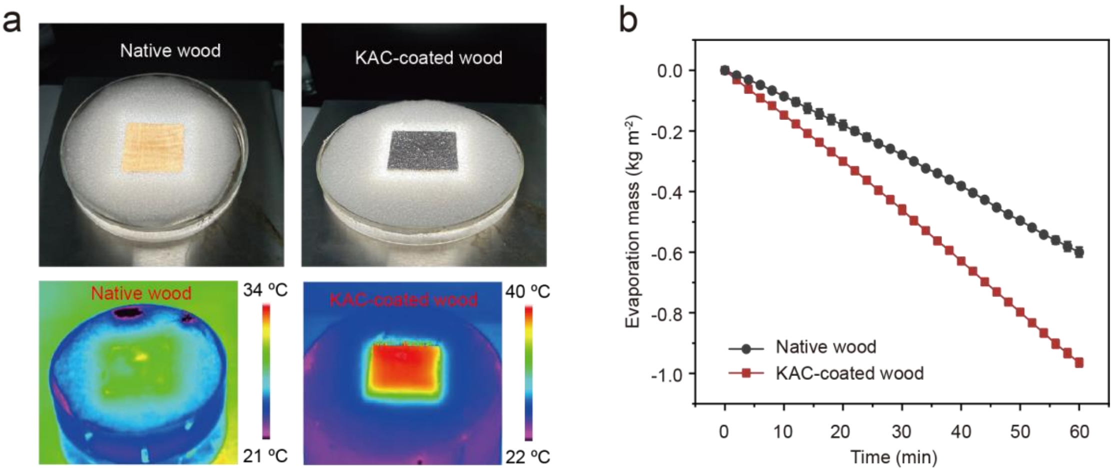

Figure S5. Photographs and related IR images (a), and water evaporation rate (b) of native wood and KAC-coated wood 2D evaporators with flat sizes of 4 cm x 4 cm under 1 kW m^{-2} solar radiation and without wind.

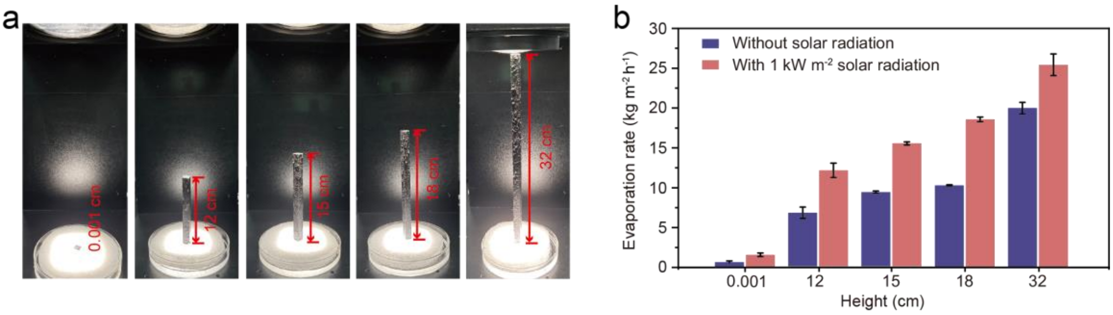

Figure S6. Photographs of KAC-coated wood evaporator with heights of 0.001, 12, 15, 18 and 32 cm with 1 kW m^{-2} solar radiation (a) and water evaporation rate of KAC-coated wood evaporator with heights of 0.001, 12, 15, 18 and 32 cm with 1 kW m^{-2} solar radiation or without solar radiation (b) at 0 m s^{-1} wind speed.

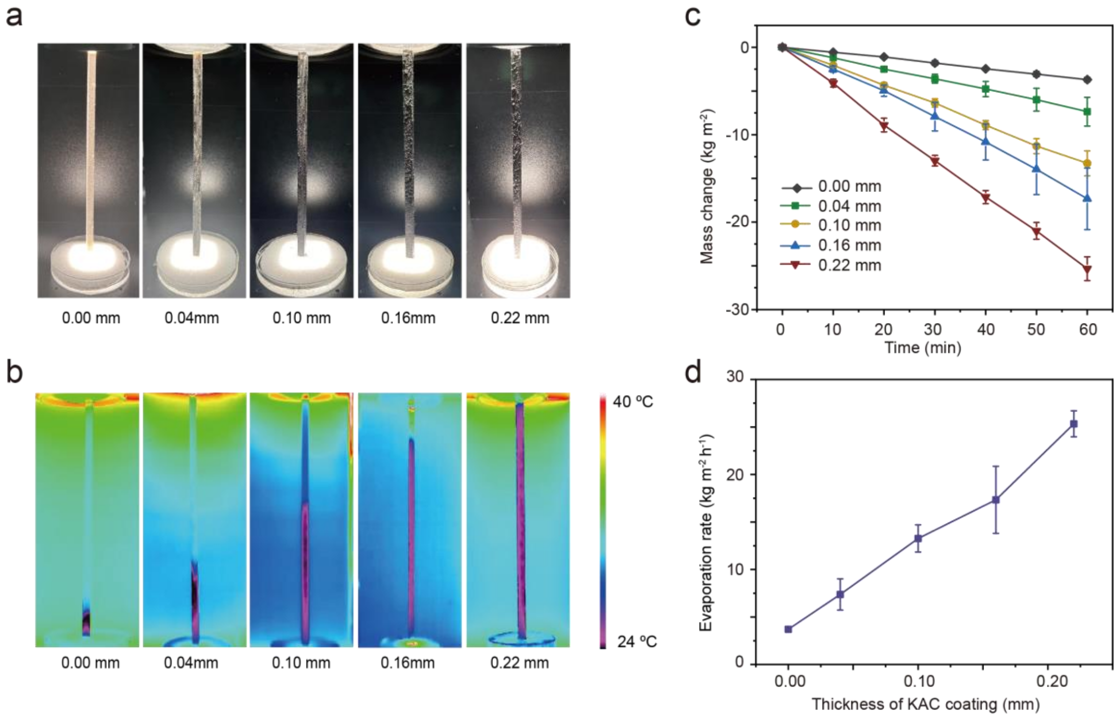

Figure S7. Photographs (a), related IR images (b), evaporation rates (c) and the correlation (d) of KAC-coated wood evaporator at height of 32 cm with various KAC coating thickness under 1kW m^{-2} solar radiation without wind.

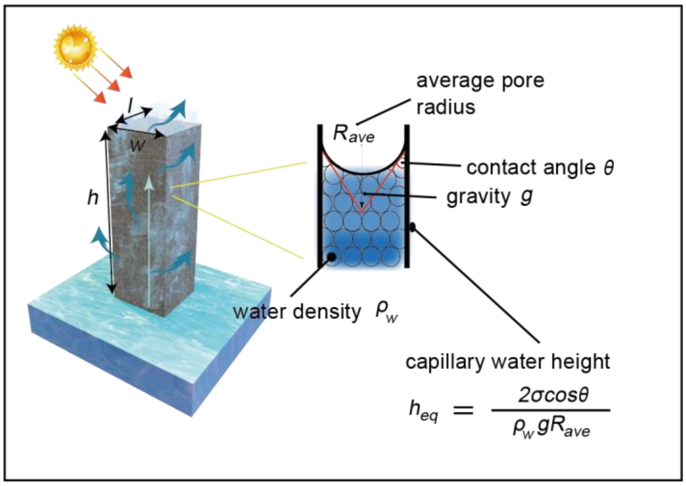

Figure S8. Schematic description of the working principle for Jurin's law.

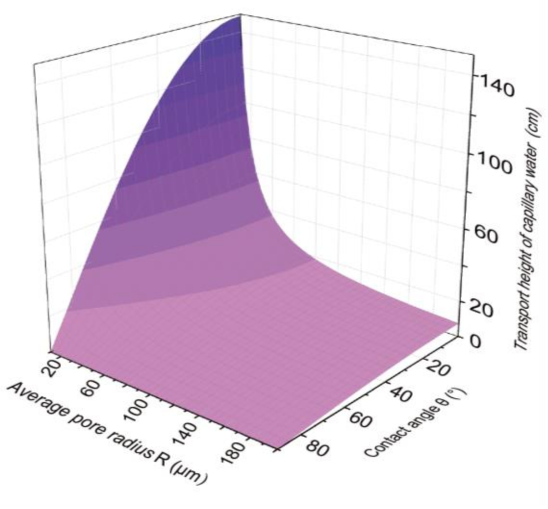

Figure S9. Theoretical estimation of the influence of contact angle and average pore radius on the equilibrium capillary transport height according to Jurin's law.

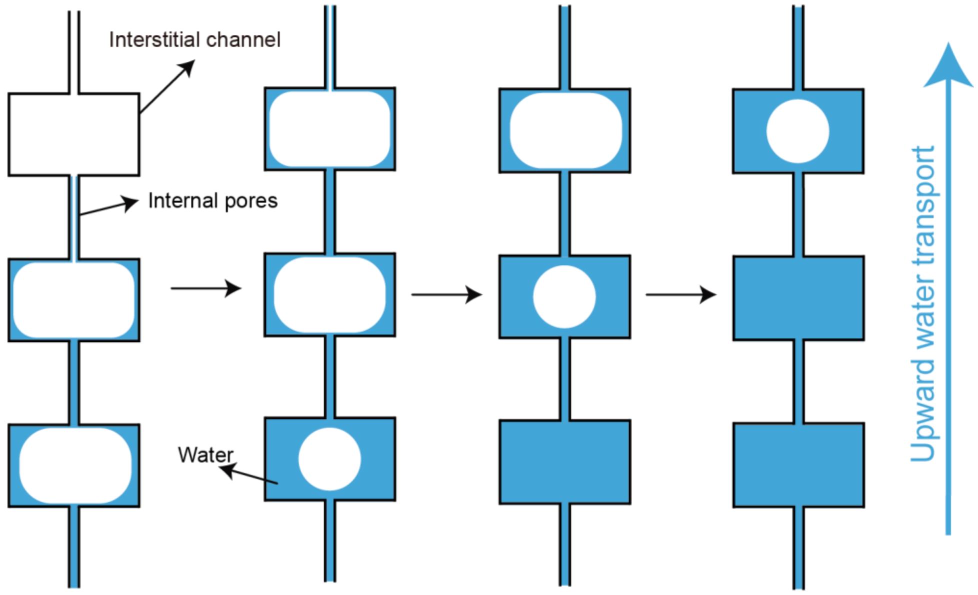

Figure S10. Schematic diagram to show the process in facilitating the upward water transport of KAC-coated wood evaporator.

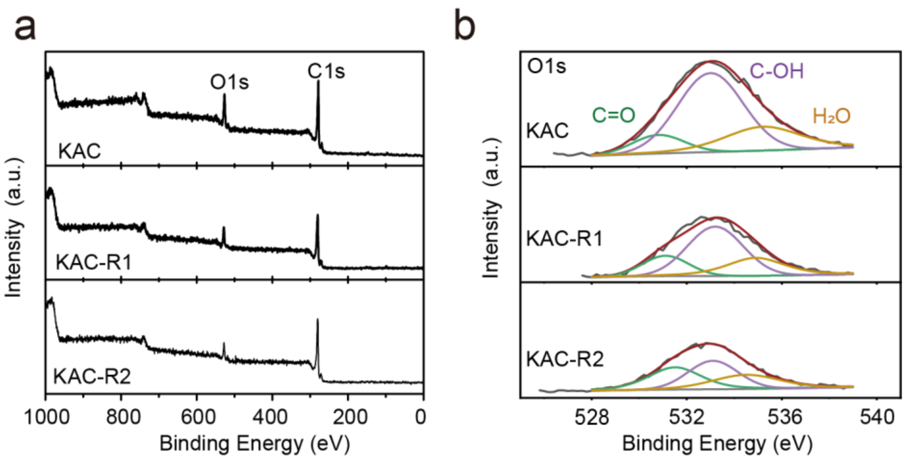

Figure S11. XPS spectra (a) and XPS spectrum of O1s (b) of KAC, KAC-R1 and KAC-R2.

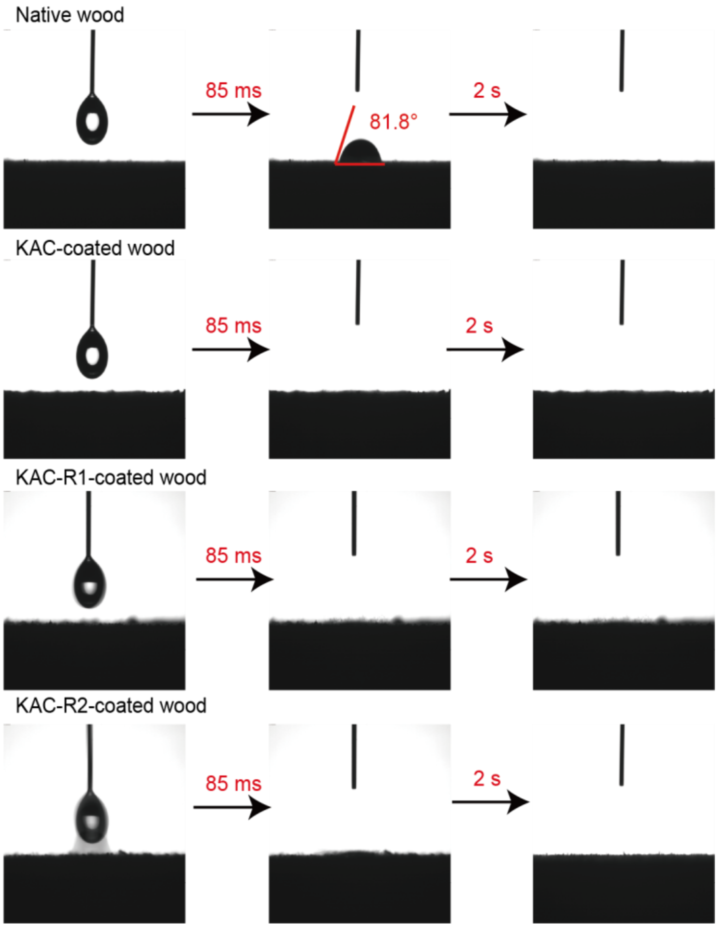

Figure S12. Water contact angle measurement of native wood, KAC-coated wood, KAC-R1-coated wood, and KAC-R2-coated wood evaporator surfaces at 85 ms and 2 s after water dropped.

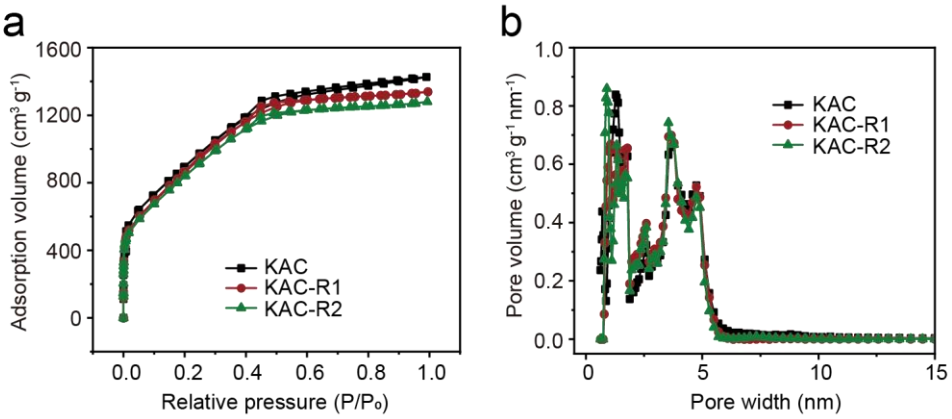

Figure S13. N_2 adsorption-desorption isotherms at 77 K (a), and pore size distributions calculated from by NLDFT method (b) of KAC, KAC-R1 and KAC-R2.

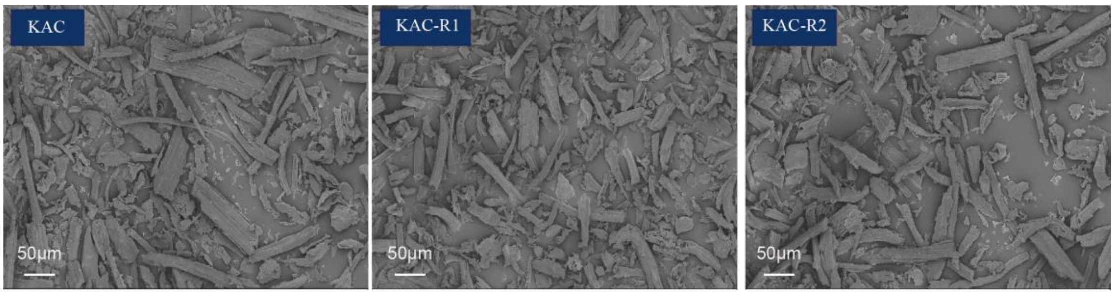

Figure S14. SEM images of KAC, KAC-R1 and KAC-R2.

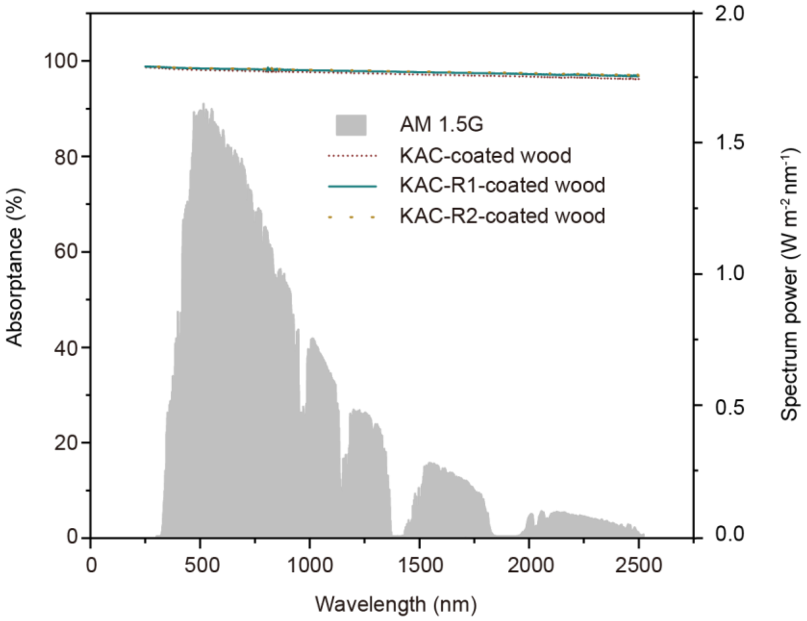

Figure S15. Solar absorbance spectra of KAC-coated wood, KAC-R1-coated wood and KAC-R2-coated wood evaporators.

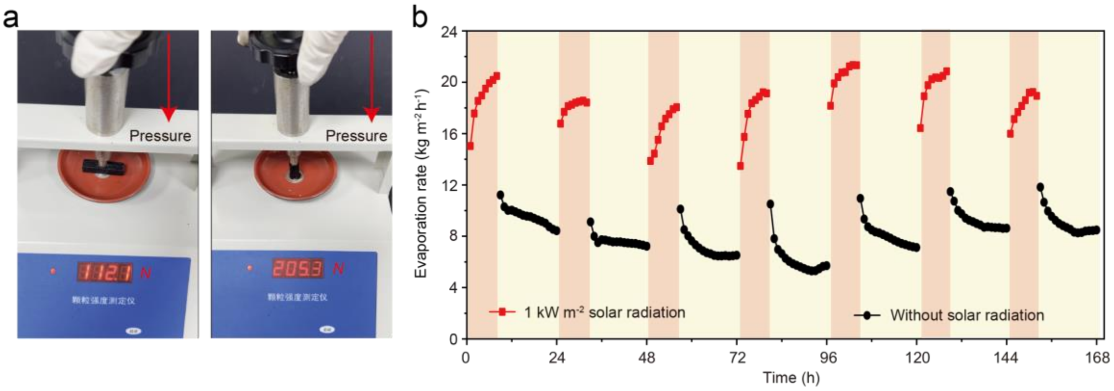

Figure S16. The maximum intensity of pressure on the transverse and longitudinal surface of KAC-coated wood (a) and the evaporation performance during a 7-day test (under 1 kW m^{-2} solar radiation for 8 h and without solar radiation for 16 h every day) of KAC-coated wood evaporator at height of 18 cm (b).

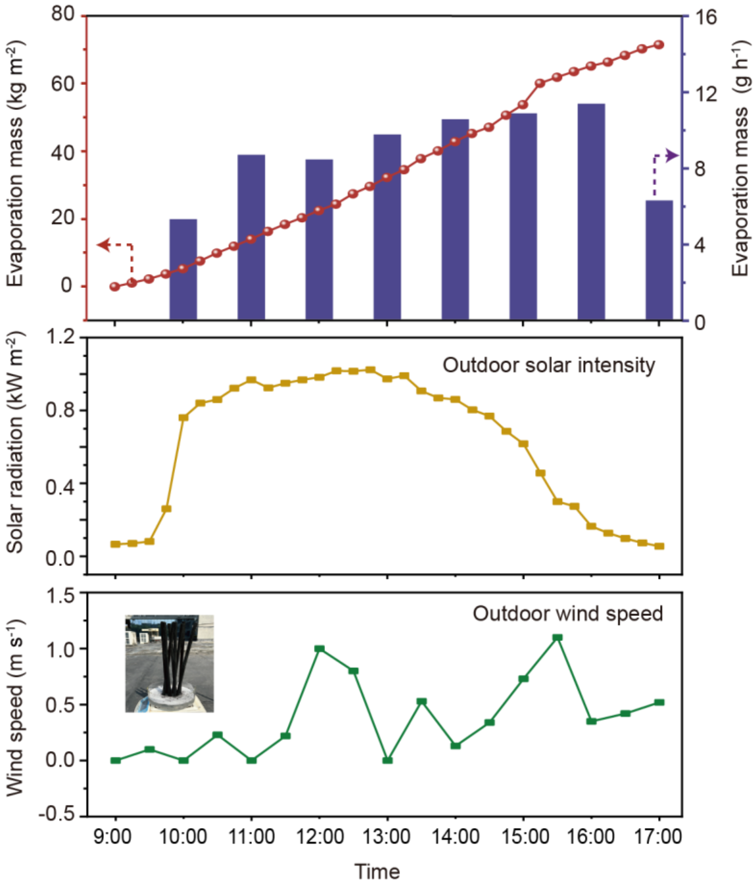

Figure S17. Outdoor steam generation experiment with array of nine KAC-coated wood evaporators.

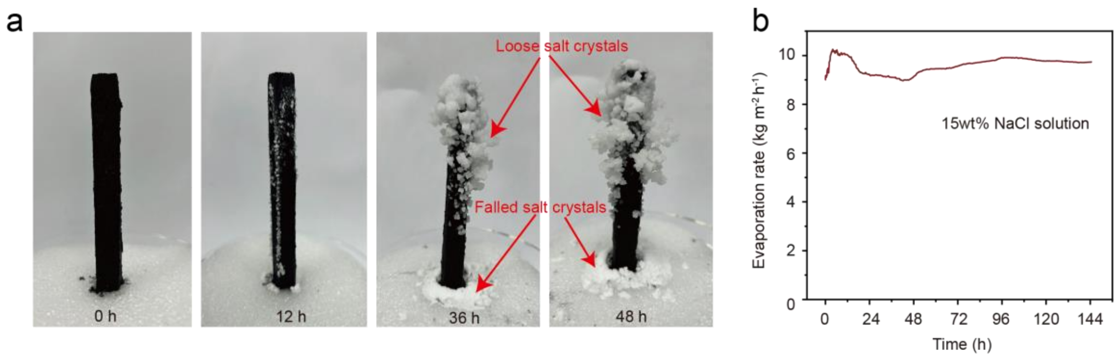

Figure S18. Photographs (a) and evaporation rate (b) of KAC-coated wood evaporator with a height of 12 cm for treating NaCl solution of 15 wt% under 1 kW m^{-2} solar radiation without wind.

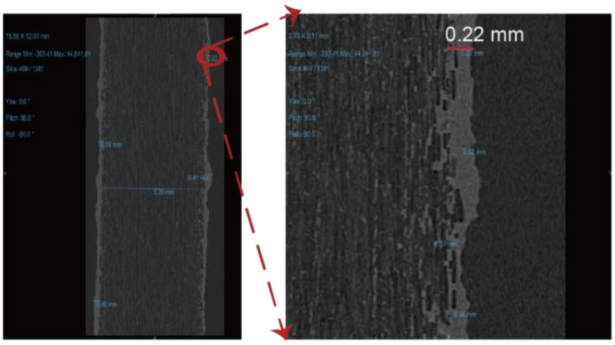

Figure S19. X-ray computed tomography measurement images to show the thickness of the KAC layer with the loaded KAC mass of 1.5 mg cm^{-2}.

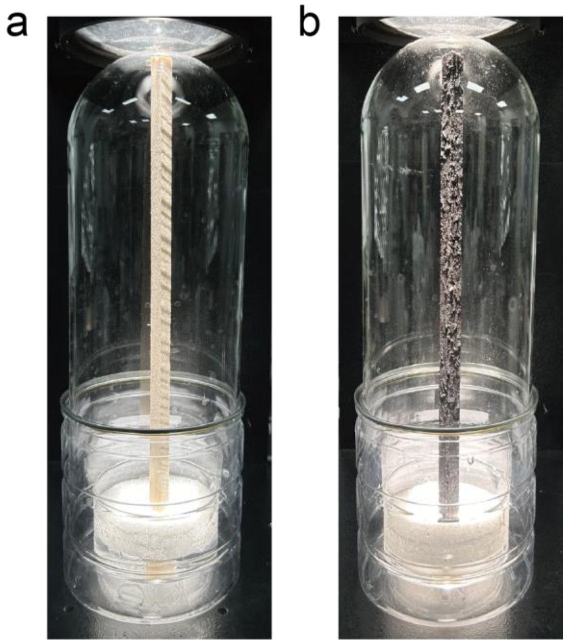

Figure S20. Photographs of evaporation-condensation device of KAC-coated wood evaporator with the height of 32 cm under 1 kW m^{-2} solar radiation and without wind.
